# Multi-Omics Analyses Reveal the Molecular Mechanisms Underlying the Adaptation of Wheat (*Triticum aestivum* L.) to Potassium Deprivation

**DOI:** 10.3389/fpls.2020.588994

**Published:** 2020-10-06

**Authors:** Yong Zhao, Ruoxi Sun, Haodong Liu, Xiaowei Liu, Ke Xu, Kai Xiao, Shuhua Zhang, Xueju Yang, Cheng Xue

**Affiliations:** State Key Laboratory of North China Crop Improvement and Regulation, Hebei Agricultural University, Baoding, China

**Keywords:** wheat, varietal diversity, ionome, transcriptome, metabolome, potassium utilization efficiency

## Abstract

Potassium (K) is essential for regulating plant growth and mediating abiotic stress responses. Elucidating the biological mechanism underlying plant responses to K-deficiency is crucial for breeding new cultivars with improved K uptake and K utilization efficiency. In this study, we evaluated the extent of the genetic variation among 543 wheat accessions differing in K-deficiency tolerance at the seedling and adult plant stages. Two accessions, KN9204 and BN207, were identified as extremely tolerant and sensitive to K-deficiency, respectively. The accessions were exposed to normal and K-deficient conditions, after which their roots underwent ionomic, transcriptomic, and metabolomic analyses. Under K-deficient conditions, KN9204 exhibited stronger root growth and maintained higher K concentrations than BN207. Moreover, 19,440 transcripts and 162 metabolites were differentially abundant in the roots of both accessions according to transcriptomic and metabolomic analyses. An integrated analysis of gene expression and metabolite profiles revealed that substantially more genes, including those related to ion homeostasis, cellular reactive oxygen species homeostasis, and the glutamate metabolic pathway, were up-regulated in KN9204 than in BN207 in response to low-K stress. Accordingly, these candidate genes have unique regulatory roles affecting plant K-starvation tolerance. These findings may be useful for further clarifying the molecular changes underlying wheat root adaptations to K deprivation.

## Introduction

Potassium (K) is a critical inorganic nutrient for plants, wherein it has vital effects on several processes, including energy metabolism, protein synthesis, and solute transport, but especially enzyme activities, stomatal movement, and the maintenance of the cation–anion balance (Zed and Paul, 2008; Amtmann et al., 2005; White and Karley, 2010; Wang et al., 2013). Many agricultural soils worldwide lack sufficient K contents, which has restricted sustainable crop development. In China, over 25% of the arable land (30 million ha) is deficient in K. The application of K fertilizers has effectively increased crop productivity in intensive cropping systems (Wu and Wang, 2015). However, the frequent and excessive use of K fertilizers has resulted in serious environmental problems, while also increasing farming costs (Laegreid et al., 1999). Therefore, further elucidating the molecular mechanisms underlying plant responses to K-starvation stress and breeding crop varieties that can more efficiently take up and use soil K are important for developing a more sustainable agricultural system.

Wheat has been cultivated in China for more than four millennia and is now grown in 10 major agro-ecological zones (Zhuang, 2003). Owing to a long evolutionary period and the artificial selection in different regions, the wheat germplasm in China exhibit regional genetic characteristics (He et al., 2001; Hao et al., 2008). Tolerance to K-deficiency is a complex quantitative trait, with strong interactions between genotypes and the environment. Additionally, there are the considerable differences in the K-deficiency tolerance among wheat species (Zhao et al., 2014). To date, there has been relatively little research conducted to characterize the K-deficiency tolerance of the available wheat germplasm at different growth stages. Therefore, an efficient characterization of wheat plants in terms of their tolerance to K-deficiency may produce important information for the development and cultivation of wheat genotypes exhibiting increased tolerance to insufficient K contents in soil.

Plant responses to K-deficiency stress are due to various complex gene regulatory networks that induce extensive changes in gene expression as well as protein and metabolite contents (Liang et al., 2013). The omics-based technologies, including ionomics, transcriptomics, proteomics, and metabolomics, have enabled high-throughput and efficient comprehensive analyses of K-deficiency stress-induced changes to ions and gene expression as well as protein and metabolite levels in plants (Hafsi et al., 2014). To date, there has been little research on ionomic responses to nutrient stress in wheat, but ionomics-based techniques are becoming powerful research tools for studying plant physiology (Wu et al., 2013; Zeng et al., 2015). Previously, the molecular mechanisms underlying plant responses to K-deficiency were dissected via transcriptomic and proteomic analyses, resulting in the identification of many K-responsive genes and proteins of *Triticum aestivum* (Ruan et al., 2015; Zeng et al., 2015; Quan et al., 2016; Li et al., 2017; Zhao et al., 2019). Additionally, metabolomics-based studies have revealed intermediate metabolites relevant to various plant physiological characteristics under different abiotic stress conditions. Zeng et al. (2018) identified 57 kinds of metabolites as well as differences in the phenylpropanoid metabolic pathway mediated by phenylalanine ammonia-lyase (PAL) among three genotypes, which may be closely associated with the genotypic differences in K-deficiency tolerance. However, there are few reports describing integrated analyses of the genes and metabolites involved in the K-deficiency stress response pathways in wheat.

This study was designed to evaluate the level of genetic variation among 543 wheat accessions (531 were Chinese wheat cultivars from 10 provinces) for K-deficiency tolerance at seedling and mature stages, with the aim to identify contrasting (K-deficiency tolerant and K-deficiency sensitive) genotypes for further breeding programs and genetic studies. The identified genotypes were further conducted ionomic, transcriptomic, and metabolomic analyses using the roots under normal or K-deficient conditions. The objectives of this study were to (1) determine the differences between the ionomes, transcriptomes, and metabolomes of two wheat accessions in response to low-K stress and (2) identify the signaling pathways and regulatory networks related to low-K tolerance, which further characterized the wheat root molecular responses enabling adaptations to K deprivation.

## Materials and Methods

### Plant Materials

A total of 543 bread wheat germplasm accessions were used for evaluation of K-deficiency tolerance. Including 488 cultivars, 43 regional test lines from the 10 major wheat-growing Provinces of China, and 12 additional non-Chinese parental lines (Zhao et al., 2020).

### Low-K Tolerance Evaluation

#### Hydroponic Test

The wheat germplasm accessions were hydroponically grown in a greenhouse at Hebei Agricultural University, Baoding, China. The plants in modified Hoagland’s solution were screened at the seedling stage in a supported hydroponic system (Guo et al., 2012). The K treatment involved two concentrations prepared with K_2_SO_4_ in nutrient solution [2.0 × 10^–3^ mol/L as the control (CK) and 0.01 × 10^–3^ mol/L for the low-K (LK) treatment]. A completely randomized design was employed, with three replicates per treatment. Seeds (300 grains per wheat germplasm accession) were sterilized in 3% H_2_O_2_ for 5 min, rinsed three times with distilled water, and immersed in distilled water for 24 h. The uniformly germinated seeds were then transferred to Petri dishes containing three layers of filter paper and then incubated in an illuminated growth chamber at 20°C. Seven days later, the endosperm was removed from 30 uniformly and vigorously growing seedlings of each genotype. For each replication, 10 plants of each genotype were transferred to 25-L containers, which were placed in an illuminated growth chamber (20°C with a 12-h light/12-h dark cycle). Additionally, the nutrient solution was aerated continuously and renewed every 3 days for the duration of the experiment at which time the pH was re-adjusted to 6.0.

Wheat seedlings were harvested after 14 days of growth in the nutrient solution. Thirty seedlings (three replicates) were rinsed with distilled water and then divided into the roots and shoots. The shoot length (SL), leaf length (LL), and leaf width (LW) were measured and the leaf area (LA) was calculated. Root and shoot samples were dried in an oven at 105°C for 30 min and then at 80°C until they were completely dry (approximately 48 h). The total dry weight (TDW), shoot dry weight (SDW), and root dry weight (RDW) were measured, after which the root-to-shoot dry weight (RSDW) ratio was calculated. The K concentration (SKCe, RKCe) was determined for the digested samples. Moreover, the K content (SKC, RKC) and K utilization efficiency (SKUE, RKUE) were calculated. Specific details regarding the methods used for analyzing samples are listed in Supplementary Table 1.

#### Pot Incubation Test

The pot experiments involving two treatment (180.00 mg/kg as the CK and 45.00 mg/kg for the LK treatment) were conducted under a movable transparent rain shelter at Hebei Agricultural University, Hebei, China. The experiments were conducted with a completely randomized design, with three replicates per treatment. Eighteen seeds were planted in plastic pots (20 cm diameter and 21 cm height) at a depth of 2 cm. The seedlings were thinned to 14 plantlets per pot at the three-leaf stage. Pots were filled with 6 kg sieved soil (0.90 g/kg organic matter, 0.17 g/kg total N, 17.95 g/kg total K, 535.00 mg/kg slowly available K, 4.18 mg/kg available P, and 45.00 mg/kg available K). The base fertilizer comprising 0.63 g urea (N 46.4%) and 0.94 g DAP (N 18.0%, P_2_O_5_ 46.0%) per pot was applied before seeding, 1.53 g K_2_SO_4_ (K_2_O 53.0%) was applied at the CK treatment. Additionally, the wheat plants in each pot were treated with 0.63 g urea during the reviving stage. Fungal diseases and insects were controlled by spraying growing wheat plants three times with a fungicide and once with the insecticide.

At the mature stage, three plants were randomly selected from each pot to measure the plant height (PH), LL, LW, and LA. After harvesting, the number of kernels per spike (KPS), SDW, 1,000-grain weight (TGW), grain yield (GY), and K concentration (SKCe, StKCe, GKCe) were determined. Moreover, the K content (SKC, StKC, GKC) and K utilization efficiency (SKUE, StKUE, GKUE) were calculated. Specific details regarding the methods used for analyzing samples are listed in Supplementary Table 1.

### Principal Component Analysis and Comprehensive Evaluation

The low-K tolerance coefficient for each index was calculated as previously described (Yuan et al., 2005). The membership function value [U (x)], weight (w), and low-K tolerance comprehensive evaluation value (D) of each genotype was calculated using published methods (Zhou et al., 2003). The low-K tolerance coefficient was calculated as follows: mean measured value for the low-K stress/mean measured value for the control.

(1) U(Xj)=Xj-XjminXjmax-Xjmin,j= 1, 2,and 3

*U*_(_*_*Xj*_*_)_ is the membership function value of the comprehensive index *j*; *X*_*j*_ is the measured value for the low-K tolerance coefficient of index *j*; *X*_*j min*_ is the minimum value for the low-K tolerance coefficient of index *j*; *X*_*j max*_ is the maximum value for the low-K tolerance coefficient of index *j*.

(2) Wj=Pj/∑j=1nPj,j= 1, 2,and 3

*W*_*j*_ is the weight of the comprehensive index *j* in all comprehensive indices; *P*_*j*_ is the contribution rate of the comprehensive index *j* of each genotype.

(3) D=∑j=1n[U(xj)×Wj],j= 1, 2,and 3

*D* is the low-K tolerance comprehensive index evaluation value.

X1–X13 respectively represent the following indices at the seedling stage: LL, LA, SL, SDW, RDW, TDW, RSDW, SKCe, RKCe, SKC, RKC, SKUE, and RKUE. Y1–Y13 respectively represent the following indices at the mature stage: PH, LL, LA, KPS, SDW, GY, TGW, StKCe, StKC, StKUE, GKCe, GKC, and GKUE.

### Multi-Omics Analysis of Wheat Under Low-K Conditions

On the basis of the results for the above experiment, genotypes tolerant and sensitive to low-K stress (KN9204 and BN207, respectively) were selected for a low-K treatment under hydroponic culture conditions. The experiment was performed in triplicate for each treatment. After a 14-day LK treatment, the plants of each genotype were harvested for phenotypic and multi-omics analyses.

#### Elemental Content Analysis of Wheat

The harvested plants were dried in an oven at 105°C for 30 min and then at 80°C until reaching a constant mass. The plant samples were ground to a powder, after which 0.20 g ground sample was added to a tube for a digestion according to the nitric acid–perchloric acid method developed by Zasoski and Burau (1977). The clear and transparent digestion solution was transferred to a 50-mL volumetric flask containing deionized water. The N and P contents were measured with the Kjeldahl method and the vanadium aluminum yellow colorimetry method, respectively, whereas the K, Na, Ca, Mg, Fe, Mn, Cu, and Zn contents were determined with an atomic absorption spectrophotometer.

#### Transcriptomic Analysis of Wheat Under Low-K Conditions

After a 14-day LK treatment, the roots from 10 plants were collected, combined, immediately frozen in liquid nitrogen, and then stored at −80°C. Three biological replicates were collected for each treatment for the subsequent analysis. Total RNA was extracted from the frozen roots with the TRIzol reagent (Invitrogen, CA, United States) following the manufacturer’s procedure. The total RNA quantity and purity were analyzed with the 2100 Bioanalyzer and the RNA 6000 Nano LabChip Kit (Agilent, CA, United States), with an RNA integrity number >7.0. Polyadenylated mRNA was purified from the high-quality total RNA with poly-T oligo-attached magnetic beads (Invitrogen). The cleaved RNA fragments were reverse-transcribed to create the final cDNA libraries with the mRNASeq sample preparation kit (Illumina, San Diego, CA, United States). The paired-end (150 bp) sequencing of the cDNA libraries was completed with the Illumina HiSeq 4000 system at LC Sciences (United States) following the vendor’s recommended protocol. Low-quality reads (e.g., poly-N sequences) and reads with adapters were removed from the raw data, and the remaining clean reads were retained for the subsequent analysis. The HISAT software was used to align clean reads to the wheat reference genome IWGSC^1^ reference genome, and the transcripts were assembled based on the sequence alignment results (Kim et al., 2015). The edgeR program was used to analyze differentially expressed genes (DEGs), which were detected based on the following criteria: | log_2_(fold-change)| ≥ 1 and *p* < 0.05. The gene ontology (GO) and the Kyoto Encyclopedia of Genes and Genomes (KEGG) pathway enrichment analyses of the DEGs were completed with GOseqR and KOBAS, respectively.

#### Metabolomic Analysis of Wheat Under Low-K Conditions

The freeze-dried roots were ground to a powder, after which 100 mg powder was treated with 1.0 mL 70% aqueous methanol overnight at 4°C. Following a centrifugation at 10,000 × *g* for 10 min, the extracts were added to a CNWBOND Carbon-GCB SPE Cartridge (250 mg, 3 mL; ANPEL, Shanghai, China) and filtered (SCAA-104, 0.22 μm pore size; ANPEL) before the UPLC-MS analysis (Chen et al., 2013).

The sample extracts were analyzed with a UPLC-ESI MS/MS system (Shim-pack UFLC SHIMADZU CBM30A system and Applied Biosystems 6500 QTRAP mass spectrometer), with the following conditions: UPLC column, Waters ACQUITY UPLC HSS T3 C18 (1.8 μm, 2.1 mm × 100 mm); solvent system, water (0.04% acetic acid): acetonitrile (0.04% acetic acid); gradient program, 95:5 (v/v) at 0 min, 5:95 (v/v) at 11.0 min, 5:95 (v/v) at 12.0 min, 95:5 (v/v) at 12.1 min, and 95:5 (v/v) at 15.0 min; flow rate, 0.40 mL/min; temperature, 40°C; injection volume, 2 μL. MS/MS analysis was completed with an Applied Biosystems 6500 Q TRAP system (Chen et al., 2013).

On the basis of the internal database MWDB (metadata database), substances were qualitatively determined according to the second-order spectral information. The isotopic signal was removed during the analysis, including the repetitive signals of K^+^, Na^+^, and NH4^+^ as well as the repetitive signal of the fragment ion, which has a relatively large molecular weight. Metabolites were quantified via the multiple reaction monitoring mode of the triple quadrupole mass spectrometer. The metabolites were identified by univariate analysis and orthogonal partial least squares discriminant analysis. The differentially abundant metabolites were screened according to the following criteria: variable importance in projection (VIP) ≥ 1 and a fold-change ≥2 or ≤0.5 (Zhao et al., 2019).

### Statistical Analysis

The analysis of variance (ANOVA), the cluster analysis and the principal component analysis (PCA) were completed with SPSS (version 25.0) (Chicago, IL, United States). Means were compared with Duncan’s multiple comparison tests with difference considered significant at *p* < 0.05.

## Results

### Analysis of Low-K Tolerance Traits at the Seedling and Mature Stages of Wheat

Low-K tolerance-related phenotypic traits of 543 wheat genotypes at the seedling and mature stages were compared under control and low-K conditions (Tables 1, 2). In the hydroponic test, low-K stress significantly decreased the SL, LL, LA, SDW, RDW, TDW, SKCe, RKCe, SKC, and RKC, but increased the SKUE and RKUE relative to the control levels. The coefficient of variation (CV) of the different traits varied from 22.60 to 52.74% under low-K conditions. Regarding the pot incubation test, low-K stress decreased the PH, LL, LA, SDW, KPS, TGW, GY, SKCe, and SKC, but increased the SKUE. The CV of the different traits varied from 9.79 to 28.53% following the low-K treatment. These results revealed the substantial variations of most traits among the 543 wheat genotypes under low-K stress conditions during the two analyzed stages.

**TABLE 1:** Phenotypic variations in the K utilization efficiency-related traits at the seedling stage.

Stage	Trait	Treatment	Mean	Max	Min	SD	CV (%)
Seedling	LL (cm)	CK	13.07	23.82	4.50	4.24	32.46
		LK	10.21	19.07	3.12	3.14	30.78
	LA (cm^2^)	CK	2.99	5.81	0.86	1.09	36.67
		LK	2.09	4.31	0.40	0.79	37.65
	SL (cm)	CK	18.01	31.67	6.14	4.81	26.71
		LK	14.89	23.93	4.56	3.37	22.60
	SDW (mg plant^–1^)	CK	21.36	39.62	6.50	5.79	27.09
		LK	17.22	29.42	4.47	4.48	26.04
	SKCe (g kg^–1^)	CK	24.44	48.27	8.27	8.38	34.31
		LK	6.93	12.16	2.22	1.87	27.01
	SKC (mg⋅plant^–1^)	CK	1.02	1.43	0.10	0.24	46.02
		LK	0.28	0.95	0.02	0.07	52.13
	SKUE (mg^2^ SDW⋅μg^–1^ SKC)	CK	0.99	2.43	0.24	0.50	50.33
		LK	2.61	5.46	0.36	1.09	41.89
	RDW (mg plant^–1^)	CK	11.35	23.95	2.48	3.77	33.20
		LK	9.18	19.10	1.69	2.53	27.54
	RKCe (g kg^–1^)	CK	7.72	19.64	1.81	4.00	51.89
		LK	3.05	8.44	0.55	1.38	45.26
	RKC (mg plant^–1^)	CK	0.08	0.27	0.01	0.04	53.79
		LK	0.03	0.09	0.01	0.01	52.74
	RKUE (mg^2^ SDW⋅μg^–1^ SKC)	CK	1.95	6.09	0.20	1.28	65.50
		LK	3.52	8.37	0.36	1.72	48.93
	TDW (mg plant^–1^)	CK	32.41	55.44	11.01	8.51	26.25
		LK	26.39	44.06	7.97	6.43	24.35

*SL, LL, LA, SDW, RDW, and TDW are shoot length, leaf length, leaf area, shoot dry weight, root dry weight, and total dry weight at the seedling stage, respectively; SKCe and RKCe are the shoot and root K concentrations at the seedling stage, respectively; SKC and RKC are the shoot and root K contents, respectively; SKUE and RKUE are the shoot and root K utilization efficiencies at the seedling stage, respectively; CK and LK refer to the control and low-K stress conditions in the screening experiments, respectively; SD is standard deviation and CV is coefficient of variation.*

**TABLE 2:** Phenotypic variations in the K utilization efficiency-related traits at the mature stage.

Stage	Trait	Treatment	Mean	Max	Min	SD	CV (%)
Mature	PH (cm)	CK	46.07	73.00	30.00	6.14	13.32
		LK	40.49	64.68	24.33	6.05	14.95
	LL (cm)	CK	10.23	19.27	6.50	1.77	17.30
		LK	8.15	16.33	4.33	1.52	18.67
	LA (cm^2^)	CK	6.71	15.03	3.88	1.48	22.05
		LK	4.33	8.92	1.41	1.16	27.04
	SDW (mg plant^–1^)	CK	16.13	25.66	8.72	2.37	14.69
		LK	13.94	19.97	8.11	2.23	15.98
	KNPS	CK	12.26	17.33	8.67	1.20	9.79
		LK	10.53	13.67	4.67	1.48	14.02
	TGW (g)	CK	37.36	85.52	7.08	7.36	19.69
		LK	33.70	75.85	5.17	6.72	19.94
	GY (g pot^–1^)	CK	6.61	10.28	0.46	1.41	21.39
		LK	5.00	8.56	0.31	1.42	28.53
	SKCe (g kg^–1^)	CK	32.00	73.11	16.57	6.92	21.62
		LK	16.32	27.02	9.29	2.53	15.52
	SKC (mg plant^–1^)	CK	0.52	1.10	0.23	0.13	25.17
		LK	0.23	0.38	0.11	0.05	20.20
	SKUE (mg^2^ SDW⋅μg^–1^ SKC)	CK	0.53	1.17	0.13	0.13	24.95
		LK	0.88	1.52	0.36	0.21	23.48

*PH, LL, LA, SDW, KNPS, TGW, and GY are height, leaf length, leaf area, shoot dry weight, number of kernels per spike, 1,000-grain weight, and grain yield at the mature stage, respectively; SKCe, SKC, and SKUE are shoot K concentration, K content, and K utilization efficiency at the mature stage, respectively; CK and LK refer to the control and low-K stress conditions in the screening experiments, respectively; SD is standard deviation and CV is coefficient of variation.*

### PCA and Comprehensive Evaluation of the Low-K Tolerance of Different Wheat Genotypes

Thirteen indices of the low-K tolerance of various wheat genotypes at the seedling and mature stages underwent a PCA (Supplementary Tables 2, 3). The cumulative contribution of five principal components at the seedling stage was 89.69% (individual contributions of 24.21, 21.85, 17.27, 16.53, and 9.83%). Five independent comprehensive indices at the seedling stage were defined as the first to fifth principal components. The cumulative contribution of six principal components at the mature stage was 82.54% (individual contributions of 21.95, 15.45, 13.78, 12.88, 10.74, and 7.74). Six independent comprehensive indicators at the mature stage were defined as the first to sixth principal components. For both the seedling and mature stages, the PCA results are summarized in Supplementary Tables 2, 3.

**TABLE 3:** Low-K tolerant genotypes identified based on the D values in more than one growth stage.

Entry name	Seedling stage	Mature stage
	
	**Tolerant**	
Kenong9204	√	√
Henong6119	√	√
Han6172	√	
Jimai38	√	
Heng7228	√	
Cangmai119		√
Henong2552		√
Henong9204		√

*The checkmark indicates whether the genotype is tolerant in the specific stage. The order of the genotypes is based on the D value rankings.*

The principal component expression of different genotypes and the standardized low-K tolerance coefficient at the seedling and mature stages along with Eqs 1–3 were used to calculate the comprehensive index evaluation value (D value). The cluster analysis indicated that 7, 50, 238, and 248 genotypes were respectively tolerant, moderately tolerant, moderately sensitive, and sensitive to low-K stress at the seedling stage (Supplementary Table 4). The mean D values at the seedling and mature stages are provided in Supplementary Figure 1. The mean D value ranged from 0.77 (tolerant genotypes) to 0.46 (sensitive genotypes) (Supplementary Figure 1A), whereas the overall mean was 0.53. At the mature stage, 116, 214, 187, and 26 genotypes were respectively tolerant, moderately tolerant, moderately sensitive, and sensitive to low-K stress (Supplementary Table 5). The mean D value ranged from 0.69 (tolerant genotypes) to 0.43 (sensitive genotypes) (Supplementary Figure 1B), with an overall mean of 0.59. Two tolerant genotypes (KN9204 and Henong6119) (D value ranking >99th percentile) and one sensitive genotype (BN207) (D value ranking <1st percentile) exhibited consistent responses to low-K stress during the two analyzed stages (Tables 3, 4).

**TABLE 4:** Low-K sensitive genotypes identified based on the D values in more than one growth stage.

Entry name	Seedling stage	Mature stage
	
	Sensitive	
Zhengyin4hao		√
Sanshumai		√
Jining6058		√
Jw1	√	
Shannongjian36-459	√	
Xu9158	√	
Pingan8hao	√	
Yanzhan4110		√
Bainong207	√	√

*The checkmark indicates whether the genotype is sensitive in the specific stage. The order of the genotypes is based on the D value rankings.*

### Phenotypic Comparison of the Contrasting Genotypes

**FIGURE 1:**
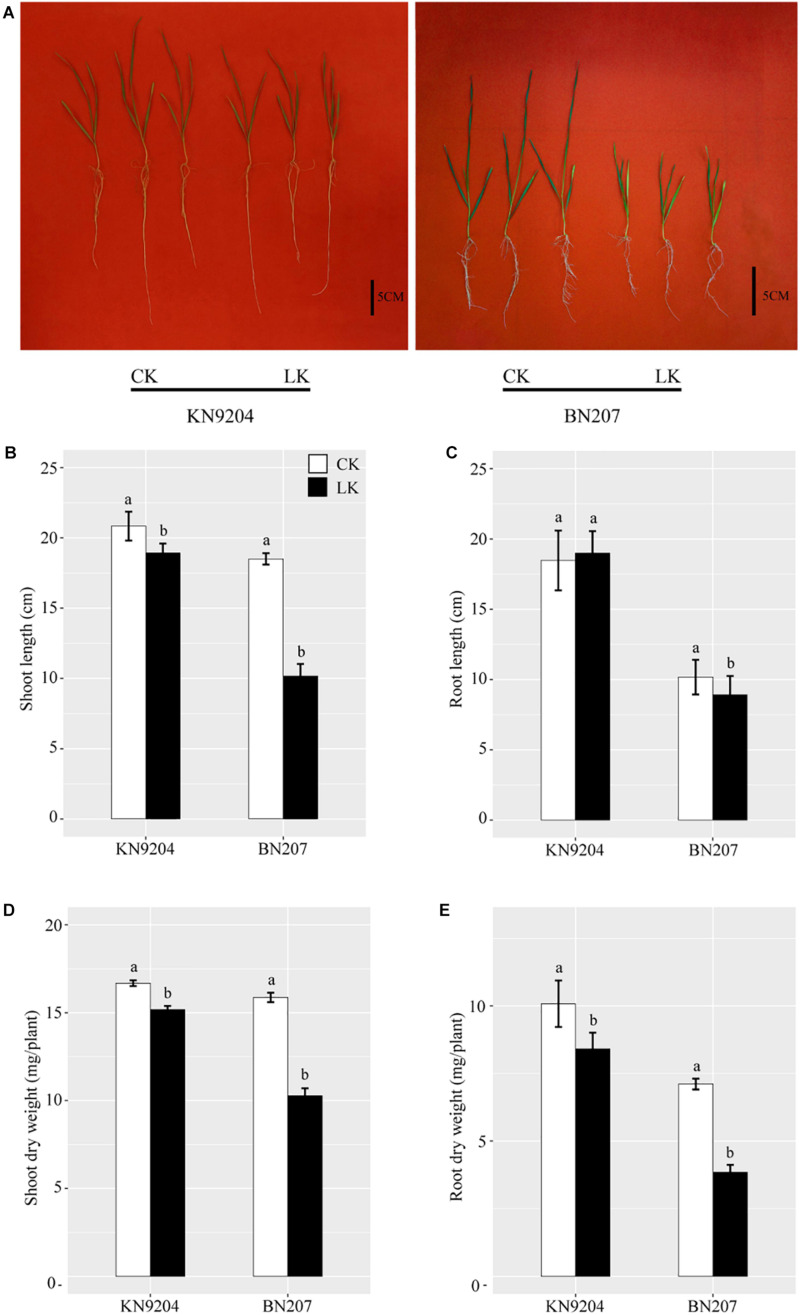
Growth performance of KN9204 and BN207 after 14 days under control (CK) and low-K (LK) stress conditions. **(A)** KN9204 plants (left) and BN207 plants (right). Bar, 5 cm. **(B)** shoot length, **(C)** root length, **(D)** shoot dry weight, and **(E)** root dry weight of KN9204 and BN207 plants. Data are presented as the mean ± SD of three biological replicates (*n* = 3). Lowercase letters indicate significant differences (*p* < 0.05) as determined by a one-way ANOVA.

A comparison between a tolerant genotype (KN9204) and a sensitive genotype (BN207) treated with different K concentrations confirmed the low-K stress tolerance varied considerably between the two genotypes (Figure 1). In response to the low-K stress treatment, the BN207 leaves were chlorotic and scorched along the margins, unlike the KN9204 leaves, which were essentially symptomless (Figure 1A). The SL of KN9204 and BN207 exposed to low-K stress decreased relative to the control values, although the decrease was less for KN9204 (Figure 1B). The RL significantly decreased under LK stress conditions in BN207, but unchanged significantly in KN9204 (Figure 1C). After 14 days of low-K stress, the SDW and RDW also decreased for both genotypes relative to the control values, but the decrease was greater for BN207 (Figures 1D,E). These observations confirmed that KN9204 is more tolerant to low-K conditions than BN207.

### Influence of Low-K Stress on the Shoot and Root Ionome of the Two Contrasting Genotypes

To reveal the effects of low-K stress on the uptake of K and other nutrients by wheat plants, the N, P, K, Ca, Mg, Na, Fe, Mn, Cu, and Zn contents in the roots and shoots were analyzed for plants that underwent a 14-day low-K stress treatment. The N, P, K, Fe, Mn, Cu, and Zn contents significantly decreased under LK stress conditions, with a greater decrease for BN207 than for KN9204 (Table 5). In response to the LK stress treatment of KN9204, the N, P, K, Fe, Mn, Cu, and Zn contents decreased. Specifically, the smallest decreases in the shoots and roots were for Mn (3.68%) and Fe (2.88%), respectively, whereas the largest decreases in the shoots and roots were for K (76.17 and 48.00%, respectively). Following the LK stress treatment of BN207, the N, P, K, Fe, Mn, Cu, and Zn contents also decreased. The smallest decreases in the shoots and roots were for Zn (6.70%) and Mn (14.35%), respectively. In contrast, the largest decreases in the shoots and roots were for K (78.87%) and P (84.23%), respectively.

**TABLE 5:** Elemental contents in different genotypes under control and low-K stress conditions.

Tissue	Treatment	Genotype	N (g/kg)	P (g/kg)	K (g/kg)	Ca (g/kg)	Mg (g/kg)	Na (g/kg)	Fe (mg/kg)	Mn (mg/kg)	Cu (mg/kg)	Zn (mg/kg)
Shoot	CK	KN9204	26.23Ab	8.59Aa	29.58Aa	3.94Aa	1.70Aa	2.58Ba	8.16Ab	9.23Aa	2.33Ab	31.83Aa
		BN207	31.82Aa	5.80Ab	26.60Ab	2.56Bb	1.55Aa	1.86Aa	18.19Aa	7.09Ab	5.82Aa	31.95Aa
	LK	KN9204	21.76Ba	7.80Aa	7.05Ba	4.64Aa	1.98Aa	2.87Aa	7.03Ab	8.89Aa	1.98Ab	30.35Ba
		BN207	16.14Bb	3.57Bb	5.62Bb	3.86Aa	1.81Aa	2.05Ab	15.04Ba	6.31Ab	4.64Aa	29.81Ba
Root	CK	KN9204	25.14Aa	4.21Aa	9.25Aa	2.63Aa	0.31Aa	2.65Aa	31.29Aa	4.58Ab	2.12Ab	35.06Aa
		BN207	23.37Ab	4.44Aa	7.00Ab	0.61Bb	0.29Aa	2.29Ba	23.77Ab	10.38Aa	6.10Aa	33.70Ab
	LK	KN9204	22.39Aa	3.40Aa	4.81Ba	2.64Aa	0.32Aa	2.80Aa	30.39Aa	3.83Ab	1.86Ab	31.09Ba
		BN207	15.07Bb	0.70Bb	1.40Bb	1.35Ab	0.31Aa	2.89Aa	19.61Bb	8.89Aa	5.12Aa	27.30Bb

*CK and LK refer to the control and low-K stress conditions in the screening experiments, respectively. All experiments were replicated three times. Different uppercase letters (A and B) indicate significant difference among the same genotype between CK and LK at the 5% level, and different lowercase letters (a and b) indicate significant difference among different genotypes under the same treatment at the 5% level.*

In contrast, the Na, Ca, and Mg contents substantially increased in the roots and shoots under LK stress conditions (Table 5), with a greater increase in BN207 than in KN9204. Under low-K stress conditions, the Ca, Mg, and Na contents increased in KN9204 shoots by 17.77, 16.47, and 11.24%, respectively, and increased in KN9204 roots by 0.38, 3.23, and 5.66%, respectively. In response to the low-K stress treatment, the Ca, Mg, and Na contents increased in the BN207 shoots by 50.78, 16.77, and 10.22%, respectively, and increased in the BN207 roots by 121.31, 6.90, and 26.20%, respectively. Thus, there were obvious differences between the contrasting genotypes regarding the elemental content changes due to LK stress. The results of the tissue ionomic analysis provided additional evidence that KN9204 is more tolerant to low-K conditions than BN207.

### Transcriptomic Analyses

#### Evaluation of the RNA-Seq Reads and Mapping Results

The sequencing of 12 libraries (four samples × three replicates) yielded approximately 50 million high-quality clean reads per library. An overview of the RNA-seq data for the 12 libraries is provided in Supplementary Table 6. The number of clean reads per library were as follows: 54,002,488 (TCK), 52,767,067 (TLK), 52,077,489 (SCK), and 53,631,401 (SLK). The proportion of the clean reads mapped to the wheat IWGSC^2^ reference genome ranged from 87.11 to 92.20%. The GC content of the clean reads was approximately 53%. These values were within the range required for high-quality mRNA libraries.

#### Identification of DEGs

**FIGURE 2:**
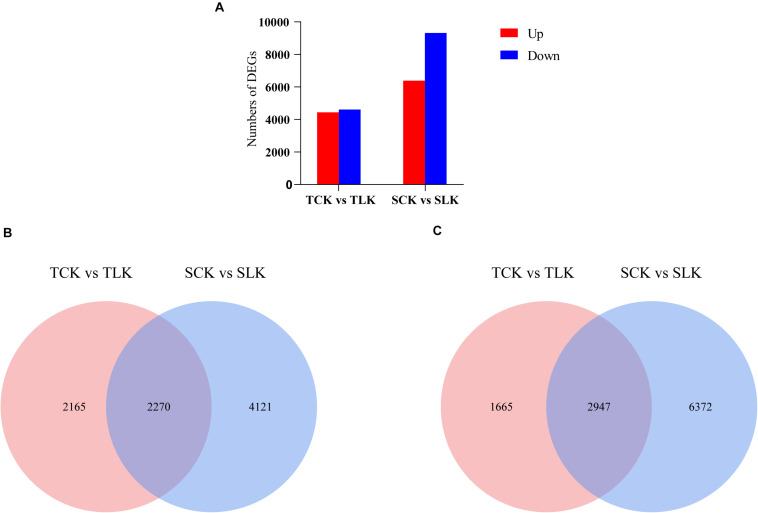
Overview of the transcriptomic analysis. **(A)** Differentially expressed genes. **(B)** Venn diagram for the up-regulated DEGs after the low-K treatment of KN9204 and BN207. **(C)** Venn diagram for the down-regulated DEGs after the low-K treatment of KN9204 and BN207. TCK, KN9204 after the control potassium treatment. TLK, KN9204 after the low-K treatment. SCK, BN207 after the control potassium treatment. SLK, BN207 after the low-K treatment.

Gene expression profiles for the wheat roots under control (normal) and low-K conditions were analyzed. An analysis of the DEGs [| log_2_(fold-change)| >1 and *p* < 0.05] revealed 4,435 up-regulated and 4,612 down-regulated DEGs in KN9204 as well as 6,391 up-regulated and 9,319 down-regulated DEGs in BN207 (Figure 2A). Additionally, 2,270 up-regulated and 2,947 down-regulated genes were common to both genotypes (Figures 2B,C).

#### DEGs Related to Transporters and CBL-Interacting Protein Kinases

In the current study, 12 and 7 DEGs encoding K transporters and K channel proteins were identified, respectively, under low-K conditions (Supplementary Table 7). The expression levels of most of the DEGs encoding K transporters were up-regulated in the two genotypes. Notably, the expression levels of three DEGs (*TraesCS2A02G116700*, *TraesCS2B02G135900*, and *TraesCS2D02G118400*) were up-regulated in KN9204, but were unchanged in BN207. The expression levels of all seven DEGs encoding K channel proteins were up-regulated, but the up-regulated expression of three of them was detected only in KN9204. Seven DEGs encoding nitrate transporters were detected, implying that nitrate transporters contribute to the metabolic activities of wheat under low-K stress conditions. Additionally, nine DEGs encoding calcineurin B-like (CBL)-interacting protein kinases (CIPKs) were identified, including six DEGs in KN9204 (all up-regulated) and six DEGs in BN207 (three up-regulated and three down-regulated). Overall, more DEGs encoding transporters and CIPKs were up-regulated in KN9204 compared to BN207.

#### DEGs Related to Oxidative Stress

We identified 179 and 11 genes encoding peroxidase and glutathione S-transferase (GST), respectively, both of which are critical for eliminating reactive oxygen species (ROS) (Supplementary Table 8). The expression of five DEGs encoding peroxidase was up-regulated in both genotypes, whereas the expression levels of 111 and 174 DEGs encoding peroxidase were respectively down-regulated in KN9204 and BN207. Moreover, We identified five up-regulated DEGs encoding GST in the two varieties, among which one gene was only up-regulated in KN9204, as well as five DEGs with down-regulated and unchanged expression levels in BN207 and KN9204, respectively (Supplementary Table 9). There were more DEGs encoding peroxidase and GST with down-regulated expression levels in BN207 than in KN9204.

#### DEGs Related to Transcription Factors

Transcription factors (TFs) are essential gene expression regulators. In this study, 430 and 758 TF DEGs were identified in KN9204 and BN207, respectively (Supplementary Table 10). These DEGs were from 35 different TF families, with more than half belonging to the CAMTA (88 in KN9204 and 104 in BN207), MYB (42 and 74), MYB_related (75 and 160), and WRKY (51 and 54) families. The DEGs also encoded members of other important TF families in plants, including bZIP, bHLH, and ERF. Among the identified TF DEGs, 409 had expression levels that were up-regulated in KN9204, but unchanged/down-regulated in BN207 or were unchanged in KN9204, but down-regulated in BN207 (Supplementary Table 11).

#### GO and KEGG Enrichment Analyses of DEGs

We selected the DEGs whose expression was significantly up-regulated in KN9204, but down-regulated/unchanged in BN207; or the DEGs whose expression was unchanged in KN9204, but down-regulated in BN207 for further analyses. Specifically, the 6,893 selected DEGs underwent a GO enrichment analysis (Supplementary Figure 2). Accordingly, the DEGs were divided into the following three main GO categories: biological process, cellular component, and molecular function. “Molecular function,” “DNA binding,” “protein binding,” and “ATP binding” were the most enriched molecular function GO terms. Regarding biological processes, “biological process,” “transcriptional regulation, DNA templating,” and “redox process” were the most enriched GO terms. The DEGs related to low-K tolerance were also associated with diverse cell components. A KEGG pathway enrichment analysis assigned these same 6,893 DEGs to 41 pathways (Supplementary Figure 3), including those related to plant hormone signal transduction, phenylpropane biosynthesis, glutathione metabolism, as well as alanine, aspartic acid, and glutamic acid metabolism.

### Metabolomic Analyses

#### Changes in the Metabolite Profiles of Two Contrasting Genotypes in Response to Low-K Stress

To clarify the differences in the metabolites of the two examined wheat genotypes under low-K stress conditions, we determined the metabolite contents of 12 samples, and detected 162 differentially abundant metabolites (Supplementary Table 12). The contents of 109 metabolites changed significantly in KN9204 (65 increased and 44 decreased). These metabolites included 20 amino acids and their derivatives, 18 lipids, 13 organic acids, and other substances. In BN207, the abundance of 91 metabolites changed significantly (29 increased and 62 decreased), including 24 organic acids, 19 amino acids and their derivatives, 15 nucleotides and their derivatives, and other substances. These metabolites are mainly involved in amino acid synthesis and metabolism, the TCA cycle, and other metabolic processes. The synthesis of most amino acids increased in KN9204 after the low-K treatment, including L-ornithine (7.12-fold), L-citrulline (5.42-fold), L-cysteine (3.9-fold), and L-(+)-lysine (3.34-fold). In contrast, decreased amino acid synthesis was detected for only tyramine (0.36-fold), L-serine (0.47-fold), and L-(−)-cystine (0.1-fold). However, in BN207, more amino acid synthesis decreased, such as L-glutamine (0.43-fold), L-glutamate (0.42-fold), and L-alanine (0.37-fold) in response to the low-K stress. Moreover, the abundances of other metabolites, such as uridine (0.35-fold) and fumaric acid (0.45-fold), decreased in BN207, but were unchanged in KN9204. Overall, the synthesis of metabolites appeared to be inhibited more in BN207 than in KN9204.

### Integration of the Transcriptomic and Metabolomic Profiles

**FIGURE 3:**
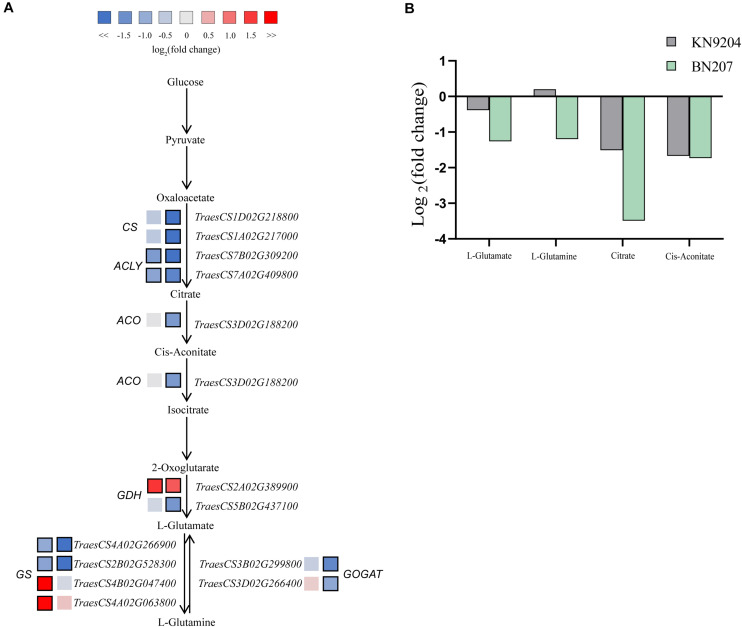
Changes in the gene expression levels and metabolite contents associated with the glutamate biosynthesis pathway of BN207 and KN9204 after the low-K treatment. **(A)** Glutamate biosynthesis pathway. Log_2_ fold-changes in DEG expression levels in KN9204 (left box) and BN207 (right box). Blue and red boxes respectively indicate the down- and up-regulated expression of the DEGs under low-K stress conditions. Bold margins indicate significant differences (*p* < 0.05) between treatments. **(B)** Changes to the metabolites in the glutamate biosynthesis pathway.

An integrated analysis of the DEGs and differentially abundant metabolites responsive to low-K stress revealed several common enriched pathways, including glutamate metabolism (Figure 3). Glutamate, which is a precursor for proline, arginine, and γ-aminobutyric acid (GABA), is important for plant growth and development. The glutamate content in BN207 decreased significantly (0.42-fold), whereas significant changes were not detected in KN9204. The contents of some metabolites related to the glutamate biosynthesis pathway decreased significantly, including citric acid (0.35-fold in KN9204 and 0.09-fold in BN207), glutamine (1.15-fold and 0.43-fold), and CIS-aconitic acid (0.31-fold and 0.30-fold). An analysis of the RNA-seq data indicated the expression levels of most of the DEGs related to the glutamate biosynthesis pathway were up-regulated or not significantly changed in KN9204, whereas the expression levels of the DEGs in BN207 were down-regulated. For example, two DEGs (*TraesCS4B02G047400* and *TraesCS4A02G063800*) encoding glutamine synthetase (GS) had up-regulated expression levels in KN9204, but relatively unchanged expression in BN207. Moreover, the expression levels of the DEGs *TraesCS3B02G299800* and *TraesCS3D02G266400*, which encode glutamate synthase (GOGAT), were significantly down-regulated in BN207, but relatively unchanged in KN9204. The results of the integrated analysis of the transcriptome and metabolome indicated that glutamate biosynthesis and metabolism were inhibited in BN207.

## Discussion

### Screening New Low-K Tolerant Genotypes Can Clarify the Complex Molecular Response of Wheat Under Low-K Stress Conditions

The availability of genetically diverse germplasm is important for the efficient breeding of new cultivars with desirable traits, including improved K uptake and K utilization efficiency. Screening new genotypes tolerant to low-K stress has enabled the identification of suitable donor parents with locally adapted genetic backgrounds in many crops (Dossougi et al., 2002; Tian et al., 2008; Wu et al., 2011). However, few studies have simultaneously evaluated low-K tolerance in more than one growth stage. In the current study, 543 wheat germplasm were evaluated regarding their tolerance to low-K stress at the seedling and mature stages. There were significant genetic differences related to the low-K tolerance traits between the germplasm at the two analyzed stages. Genotypes tolerant to low-K conditions may be used to breed for improved K utilization efficiency as well as for clarifying the genetic basis of wheat responses to K-deficiency stress. High-throughput omics-based techniques have been used to investigate complex molecular responses underlying low-K tolerance in crops (Ma et al., 2012; Fan et al., 2014; Ruan et al., 2015; Zeng et al., 2015; Quan et al., 2016; Li et al., 2017). However, no related study has been conducted to reveal the genotypic differences in wheat molecular responses to low-K stress. In the present study, we compared the ionomes, transcriptomes, and metabolomes of KN9204 (tolerant to low-K stress) and BN207 (sensitive to low-K stress) treated with different K concentrations.

### Cooperative Uptake and Interactions of K-Cations Under Low-K Stress Conditions

Ion-related physiological and biochemical activities in different tissues change considerably during wheat growth and development. The available research regarding ionomic responses to low-K stress in different wheat genotypes is limited. Therefore, to investigate the mechanisms associated with K-cation interactions in wheat under low-K conditions is very important. Under K-deprivation conditions, K (osmoticum) in mature cells can be replaced by metabolites (sugars, organic acids, and compatible solutes), and other cations can assume the role of K^+^ in the balancing of the vacuolar charge (White and Karley, 2010; Zhao et al., 2014). In the current study, we analyzed ten cations in the shoots and roots of two wheat genotypes that differed regarding their tolerance to low-K stress. The Ca, Mg, and Na contents increased significantly in the shoot and root tissues in response to low-K stress. These observations imply these cations can replace K to maintain specific physiological and biochemical activities. In contrast, the N, P, K, Fe, Mn, Cu, and Zn contents decreased significantly under low-K stress conditions, with a greater decrease in BN207 than in KN9204. Therefore, the high tolerance of KN9204 to low-K stress may be due to the active uptake and accumulation of K and other nutrients under low-K conditions.

### The Expression Patterns of DEGs in the Two Genotypes Contribute to the Differences in the K Absorption Capacity Under Low-K Stress Conditions

Plant cells absorb K mainly through K transporters and K channels, which belong to high- and low-affinity uptake systems, respectively (Zeng et al., 2014). Several K transporters and channels have been functionally characterized in plants (Amtmann and Armengaud, 2009; Dreyer and Uozumi, 2011; Wang and Wu, 2013). Previous studies proved that K transporters and K channels help plants resist the adverse effects of K-deficiency (Xu et al., 2006; Pyo et al., 2010). In this study, the expression levels of 11 DEGs encoding K transporters and seven DEGs encoding K channels were up-regulated by low-K stress. However, the expression of some of these DEGs was up-regulated in KN9204, but was unchanged in BN207. Therefore, we speculate that KN9204 can absorb K under low-K conditions better than BN207, thereby helping to explain its greater low-K tolerance.

The CIPKs are plant-specific serine/threonine protein kinases that form protein complexes with the calcium sensor CBL protein (Shi et al., 1999; Liu et al., 2000). In Arabidopsis, CIPK23 interacts with CBL1 and CBL9 to activate AKT1-mediated K^+^ uptake in roots under low-K^+^ conditions (Lee et al., 2007). Another study confirmed that CIPK6 interacts with CBL4 to regulate the transfer of the K^+^ channel AKT2 to the plasma membrane, where CIPK6 also enhances the AKT2 activity (Held et al., 2011). In the present study, the expression levels of three CIPK-encoding DEGs (*CIPK14*, *CIPK9*, and *CIPK27*) were up-regulated in KN9204, but were unchanged in BN207. Additionally, the expression of three other DEGs (*CIPK19*, *CIPK15*, and *CIPK29*) was down-regulated in BN207, but was unchanged in KN9204. We speculate that these different expression patterns of DEGs encoding CIPK between the two genotypes may contribute to the observed difference in low-K tolerance.

Low-K stress leads to excessive ROS accumulation, which can affect the functions of proteins, lipids, and even nucleic acids, leading to cell damage and even death (Mittler, 2017). Peroxidase and GST are essential for regulating ROS production and minimizing oxidative stress in plants. Armengaud et al. (2004) reported that the expression of *ATP19a*, which belongs to the peroxidase gene family, is significantly down-regulated in Arabidopsis roots under low-K stress conditions. Alka et al. (2017) observed that the expression of a GST-encoding gene is up-regulated in rice seedlings after a low-K treatment. In the current study, the expression levels of five peroxidase-related DEGs were up-regulated in the two genotypes, but the expression of the other DEGs encoding peroxidases was down-regulated. There were more DEGs with down-regulated expression in BN207 than in KN9204. Additionally, the expression of five GST-encoding DEGs was down-regulated in BN207, whereas it did not change significantly in KN9204. The differences in the peroxidase- and GST-related DEGs may enable KN9204 to maintain ROS homeostasis better than BN207, likely accounting for some of the genotypic difference in low-K tolerance.

Transcription factors bind to *cis*-regulatory elements in gene promoters to regulate expression (Lee and Young, 2000). Additionally, MYB is one of the largest TF families in plants, with crucial functions in plant stress responses. Du et al. (2019) demonstrated that MYB59 is responsive to low-K stress and mediates root-to-shoot K^+^/NO3^–^ transport by regulating the expression of *NPF7.3* in Arabidopsis roots. In this study, 35 TF families responded to low-K stress, especially the MYB TFs. Therefore, we speculate that MYB TFs have key functions related to wheat resistance to low-K stress. The underlying molecular mechanism remains to be thoroughly characterized.

### Many Metabolic Processes Are Responsive to Low-K Stress

**FIGURE 4:**
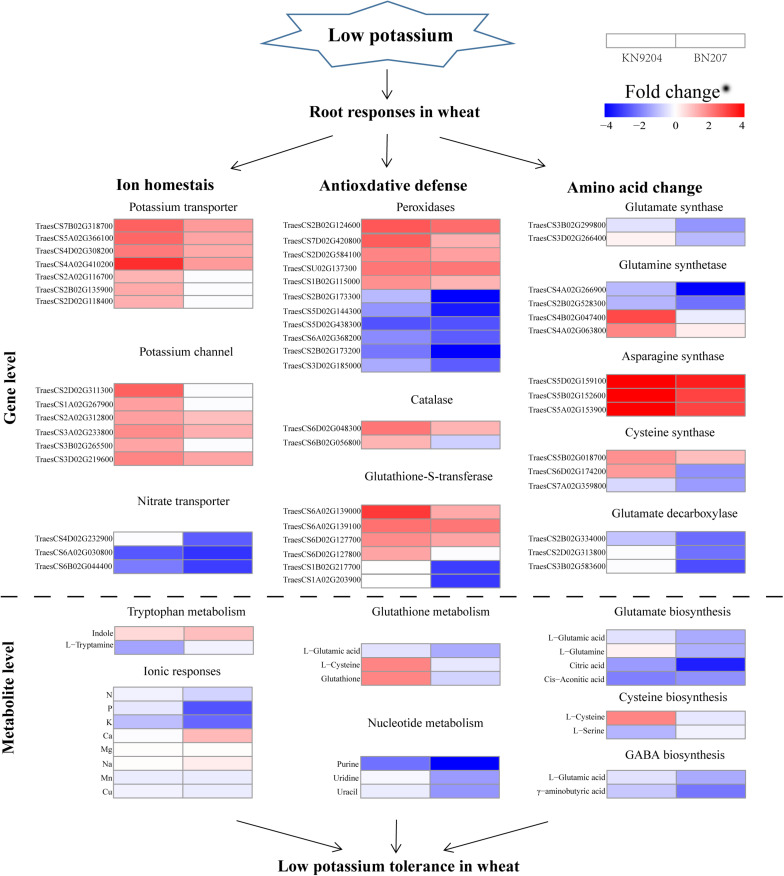
Comparison of the transcriptomic and metabolomic profiles of the KN9204 and BN207 roots after the low-K stress treatment. Genes and metabolites involved in metabolic processes are classified into three main categories: ion homeostasis, antioxidative defense, and amino acid changes. Blue indicates decreased gene expression and metabolite contents, whereas red indicates increased gene expression and metabolite contents.

Because K serves as a co-factor for enzymatic reactions and as a counter-ion for metabolite transport, K-deficiency can easily lead to metabolic disorders in plants (Armengaud et al., 2009). Armengaud et al. (2004) revealed that a low-K treatment of Arabidopsis alters carbohydrate metabolism, the TCA cycle, amino acid metabolism, and organic acid metabolism to varying degrees. In this study, we detected changes to many metabolites after a low-K treatment of wheat. The abundance of most analyzed amino acids increased in both genotypes, but some amino acid contents (e.g., L-glutamic acid, L-alanine, and GABA) decreased in BN207, but were relatively unchanged in KN9204. Our analyses also indicated that some organic acid contents decreased significantly in BN207, but not in KN9204 (e.g., D-galacturonic acid and fumaric acid). Moreover, the contents of some metabolites related to oxidative stress, such as glutathione, increased in KN9204, but did not change in BN207 (Figure 4). Glutathione is a key water-soluble antioxidant that scavenges ROS via the GSH-ascorbate cycle (Zagorchev et al., 2013). Our findings suggest that KN9204 may be able to cope with the metabolic disorders caused by low-K stress better than BN207.

### Glutamic Acid Metabolism Influences Wheat Resistance to Low-K Stress

The transcriptomic and metabolomic analyses in this study revealed substantial changes to gene expression and glutamate metabolites. Glutamate is a precursor of many stress-adaptive metabolites, such as proline, arginine, and GABA, and it is also involved in nitrogen assimilation (Forde and Lea, 2007). Low-K stress inhibits nitrogen assimilation (Hu et al., 2017), which in turn affects the synthesis of amino acids and proteins. In the current study, the glutamate content decreased in BN207 exposed to low-K stress, whereas there was no significant change in KN9204. A similar trend was observed for glutamine, which is involved in glutamate metabolism. Glutamate and glutamine are important metabolites for plant nitrogen assimilation. Thus, decreases in the glutamate and glutamine contents in BN207 may have inhibited the nitrogen assimilation process in this genotype. The down-regulated expression of the genes encoding GS and GOGAT is consistent with this hypothesis. Moreover, there were no significant changes in the glutamate and glutamine contents in KN9204. We believe that the metabolites in the glutamine metabolic pathway and the changes in the expression of the related DEGs may be key factors explaining the difference in the low-K stress tolerance between the two analyzed wheat genotypes.

In conclusion, we evaluated the genetic variations among 543 wheat accessions differing in low-K tolerance at the seedling and adult plant stages. Accessions KN9204 and BN207, were identified as tolerant and sensitive to K-deficiency, respectively. On the basis of the ionomic, transcriptomic, and metabolomic analyses presented herein, we predicted that the low-K tolerance of KN9204 is mediated by enhancements to ion homeostasis, the antioxidant defense system, and the GS signaling pathway, all of which are conducive to strong root growth and the maintenance of high K concentrations under low-K stress conditions. These findings may be relevant for clarifying the molecular responses of wheat roots to K deprivation.

## Data Availability Statement

The datasets presented in this study can be found in online repositories. The names of the repository/repositories and accession number(s) can be found below: https://www.ncbi.nlm.nih.gov/, GSE155319.

## Author Contributions

YZ and XY conceived the project and set the scientific objectives. YZ, RS, HL, KXu, XL, and SZ conducted the experiments and analyzed the data. YZ and RS wrote the manuscript. CX and KXi revised the manuscript. All authors discussed the results as well as read and approved the final manuscript for publication.

## Conflict of Interest

The authors declare that the research was conducted in the absence of any commercial or financial relationships that could be construed as a potential conflict of interest.

## Footnotes

1 http://www.wheatgenome.org/

2 http://www.wheatgenome.org/

## Supplementary Material

Supplementary Figure 1 Graphical representation of the studied genotypes based on the D value rankings. **(A)** D values of all 543 genotypes at the seedling stage. **(B)** D values of all 543 genotypes at the mature stage. The dotted line represents the mean D value for the entire population.

Supplementary Figure 2 GO cluster analysis of DEGs.

Supplementary Figure 3 Enriched KEGG pathways among the DEGs.

Supplementary Table 1 Summary of the analyzed traits and how they were examined.

Supplementary Table 2 Principal component analysis at the seedling stage.

Supplementary Table 3 Principal component analysis at the mature stage.

Supplementary Table 4 D value rankings for 543 genotypes at the seedling stage.

Supplementary Table 5 D value rankings for 543 genotypes at the mature stage.

Supplementary Table 6 Summary of sequencing data quality. TCK, KN9204 after the control K treatment; TLK, KN9204 after the low-K treatment; SCK, BN207 after the control K treatment; SLK, BN207 after the low-K treatment.

Supplementary Table 7 Genes encoding transporters and CIPKs that were differentially expressed between genotypes under low-K stress conditions. “–” indicates no significant difference in gene expression.

Supplementary Table 8 Genes encoding peroxidases that were differentially expressed between genotypes under low-K stress conditions. “–” indicates no significant difference in gene expression.

Supplementary Table 9 Genes encoding glutathione S-transferase that were differentially expressed between genotypes under low-K stress conditions. “–” indicates no significant difference in gene expression.

Supplementary Table 10 Transcription factors (TFs) identified among the differentially expressed genes.

Supplementary Table 11 Transcription factors (TFs) that satisfied the screening criteria. The screening criteria was as follows: up-regulated in KN9204, but unchanged/down-regulated in BN207 or unchanged in KN9204, but down-regulated in BN207. “–” indicates no significant difference in gene expression.

Supplementary Table 12 Fold-changes in the contents of detected metabolites in the roots of the two wheat genotypes under low-K stress conditions. “–” indicates no significant difference in content.
