# Supplementary figures and images for: Multi-Omics Analyses Reveal the Molecular Mechanisms Underlying the Adaptation of Wheat (Triticum aestivum L.) to Potassium Deprivation
Source: Front Plant Sci. 2020 Oct 6;11:588994. doi: 10.3389/fpls.2020.588994 (PMC7573229; doi:10.3389/fpls.2020.588994)

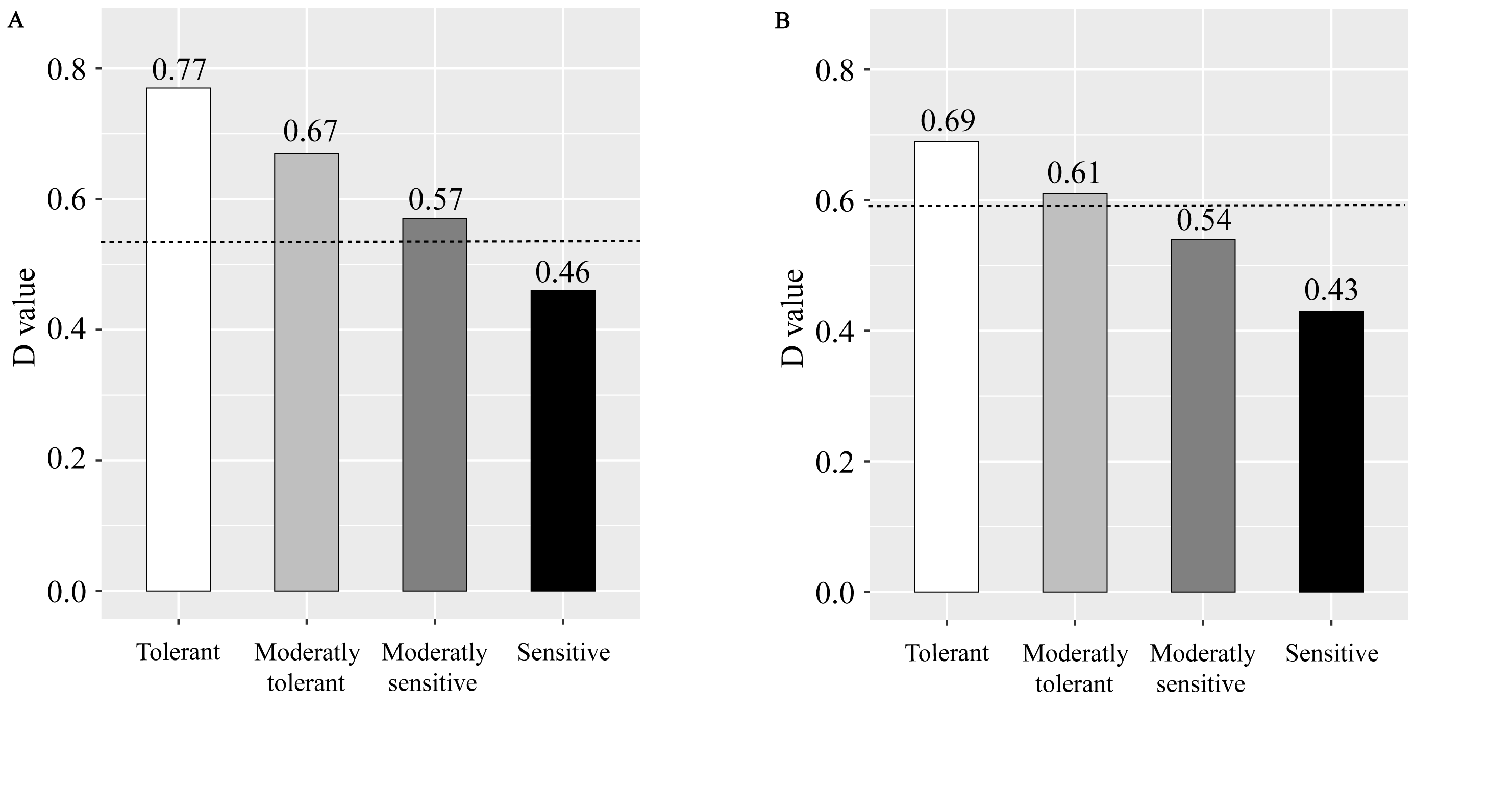

Supplement: Supplementary Figure 1 — Graphical representation of the studied genotypes based on the D value rankings. (A) D values of all 543 genotypes at the seedling stage. (B) D values of all 543 genotypes at the mature stage. The dotted line represents the mean D value for the entire population. [file Image_1.TIF]

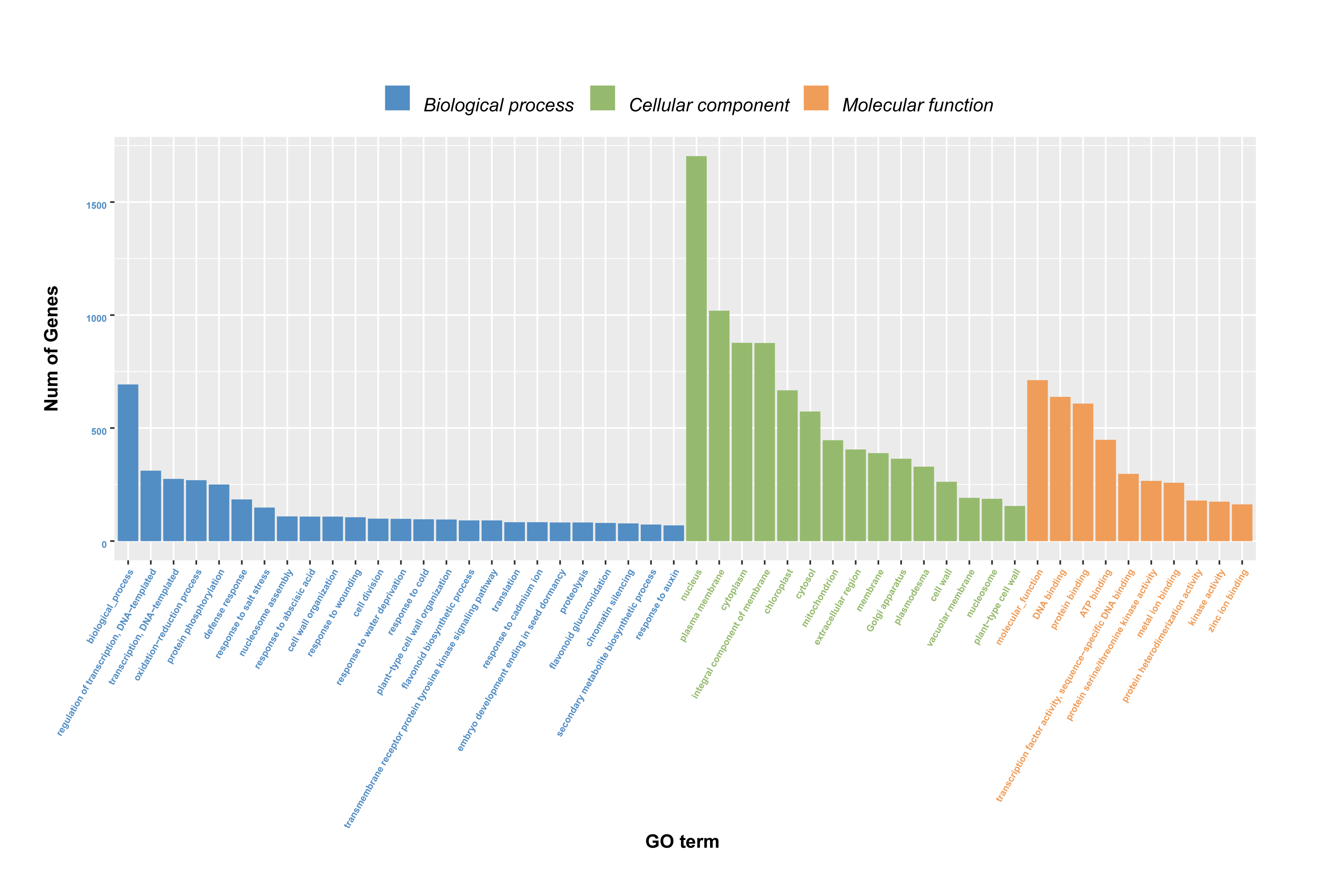

Supplement: Supplementary Figure 2 — GO cluster analysis of DEGs. [file Image_2.TIF]

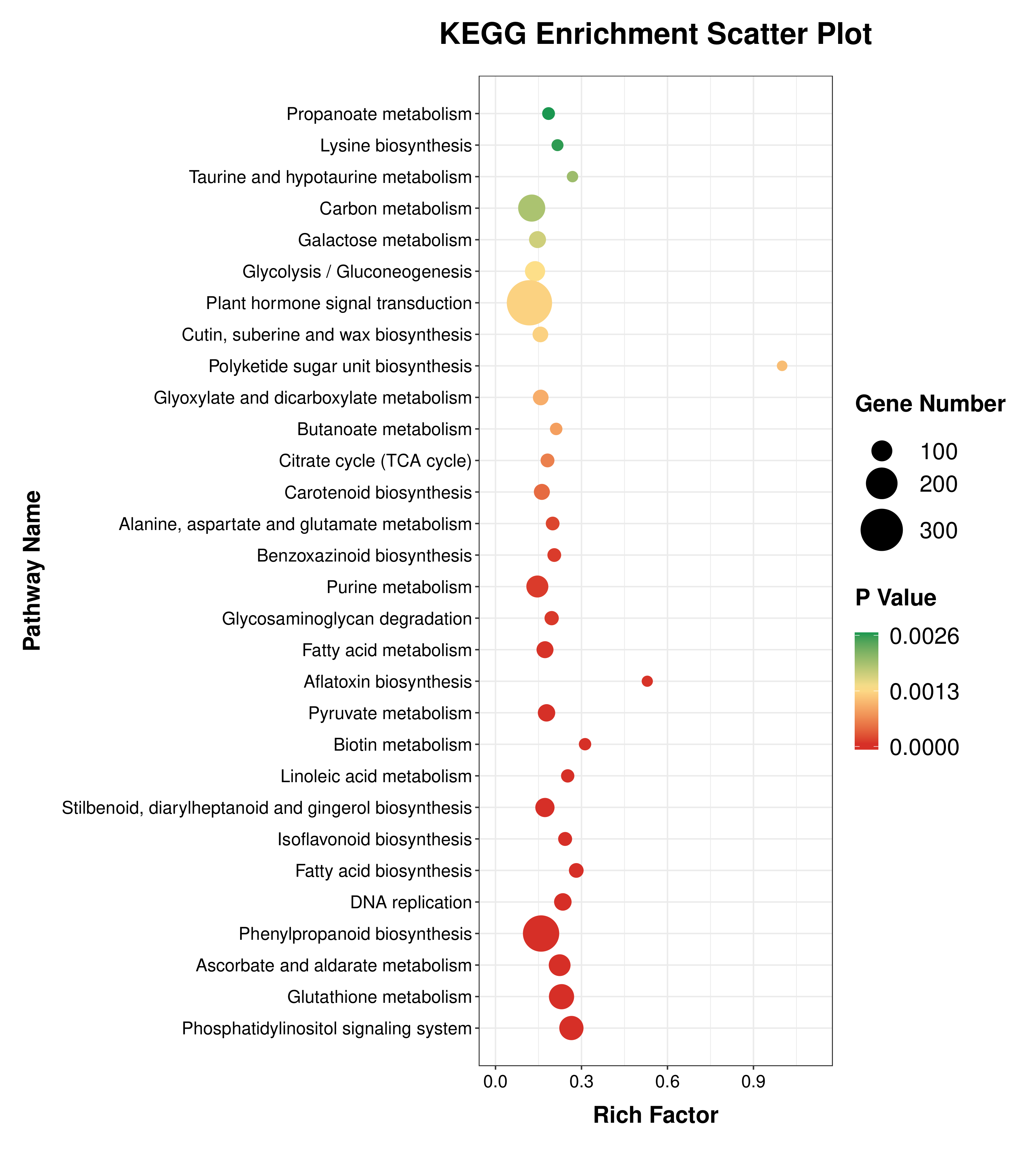

Supplement: Supplementary Figure 3 — Enriched KEGG pathways among the DEGs. [file Image_3.TIF]
